# “Fuel for the Damage Induced”: Untargeted Metabolomics in Elite Rugby Union Match Play

**DOI:** 10.3390/metabo11080544

**Published:** 2021-08-17

**Authors:** James F. Hudson, Marie M. Phelan, Daniel J. Owens, James P. Morton, Graeme L. Close, Claire E. Stewart

**Affiliations:** 1 Research Institute for Sport and Exercise Science, Liverpool John Moores University, Liverpool L3 3AF, UK; J.F.Hudson@2017.ljmu.ac.uk (J.F.H.); d.j.owens@ljmu.ac.uk (D.J.O.); j.p.mprton@ljmu.ac.uk (J.P.M.); g.l.close@ljmu.ac.uk (G.L.C.); 2 NMR Metabolomics Shared Research Facility, Technology Directorate, University of Liverpool, Crown Street, Liverpool L69 7ZB, UK; Marie.Phelan@liverpool.ac.uk

**Keywords:** metabolomics, rugby, exercise, damage, collision

## Abstract

The metabolic perturbations caused by competitive rugby are not well characterized. Our aim is to utilize untargeted metabolomics to develop appropriate interventions, based on the metabolic fluctuations that occur in response to this collision-based team sport. Seven members of an English Premiership rugby squad consented to provide blood, urine, and saliva samples daily, over a competitive week including gameday (GD), with physical demands and dietary intake also recorded. Sample collection, processing and statistical analysis were performed in accordance with best practice set out by the metabolomics standards initiative employing 700 MHz NMR spectroscopy. Univariate and multivariate statistical analysis were employed to reveal the acute energy needs of this high intensity sport are met via glycolysis, the TCA cycle and gluconeogenesis. The recovery period after cessation of match play and prior to training recommencing sees a re-entry to gluconeogenesis, coupled with markers of oxidative stress, structural protein degradation, and reduced fatty acid metabolism. This novel insight leads us to propose that effective recovery from muscle damaging collisions is dependent upon the availability of glucose. An adjustment in the periodisation of carbohydrate to increase GD+1 provision may prevent the oxidation of amino acids which may also be crucial to allay markers of structural tissue degradation. Should we expand the ‘Fuel for the work required’ paradigm in collision-based team sports to include ‘Fuel for the damage induced’?

## 1. Introduction

Rugby union is a team sport played by 9.6 million people across 159 registered unions worldwide. Rugby comprises intermittent, high intensity activities incorporating high speed running, sprinting, and frequent accelerations, and decelerations [1,2,3]. The combination of these mechanical stressors with the frequent collision-based activities can result in exercise induced muscle damage (EIMD) and impact induced muscle damage (IIMD) with distinct aetiologies [4].

The observation that total energy expenditure (TEE) was increased in young rugby players, in training weeks involving collisions [5], led to our recent investigation into how resting metabolic rate (RMR) fluctuates in elite athletes throughout a competitive match week [6]. We demonstrated that both RMR and carbohydrate oxidation in the fasted state increased significantly in the days following elite rugby union match play and proposed this was due to the muscle damage caused by the collisions inherent with the sport [6]. Despite the reports of individual metabolite fluctuations, TEE and RMR around rugby match play, our understanding of the metabolic perturbations caused by competitive rugby are not well characterized. The complex and integrated nature of the whole body exercise response means the use of metabolomics as an unbiased systems approach may be appropriate to fill the critical gaps in our understanding [7]. Recent insights from exercise metabolome studies were described in a systematic review from Schranner and colleagues [8]. A total of 196 metabolites significantly changed within 24 h of a bout of endurance or resistance exercise in human blood, sweat, urine, and saliva [8]. Significantly altered metabolites in blood samples after these exercise bouts mapped to alterations in energy production, amino acid metabolism, and indicators of oxidative stress [9,10]. 

Blood sampling in any athlete population is challenging and so addressing the utility of the minimally invasive body fluids urine and saliva should be investigated for future use in these populations [11,12]. Significant challenges remain in determining the suitability of such easily accessible biofluids, and it has been warned that the concentration of biomarkers in saliva and blood cannot be used interchangeably [13]. Whilst the comparison of lactate levels for example, in both biofluids is better in trained athletes, they do exhibit a differing response to maximal exercise [14].

Nevertheless, saliva and urinary metabolome analyses have been employed to investigate performance testing in soccer players [15], pre- and post-match play [16], and during a season to examine fatigue [17] or physical load [18] as well as in basketball throughout match play [19]. Recent work in youth soccer analyzed blood plasma, urine, and saliva to observe the effect of short-term physical activity upon the metabolome [20]. In addition to the synchronous analysis of multiple biofluids, it is also crucial to investigate the recovery period beyond 24 h as no exercise metabolomics research has examined this to date [8]. It is also paramount in this rugby population because the secondary muscle damage, experienced as delayed onset muscle soreness (DOMS), and accompanied by inflammation and satellite cell activity, peaks between 24–48 h post-match [21]. Previous work investigating inflammatory cell signaling molecules and immunoendocrine responses have gained insight measuring at these extended timepoints [22,23,24,25,26]. 

**Figure 1:**
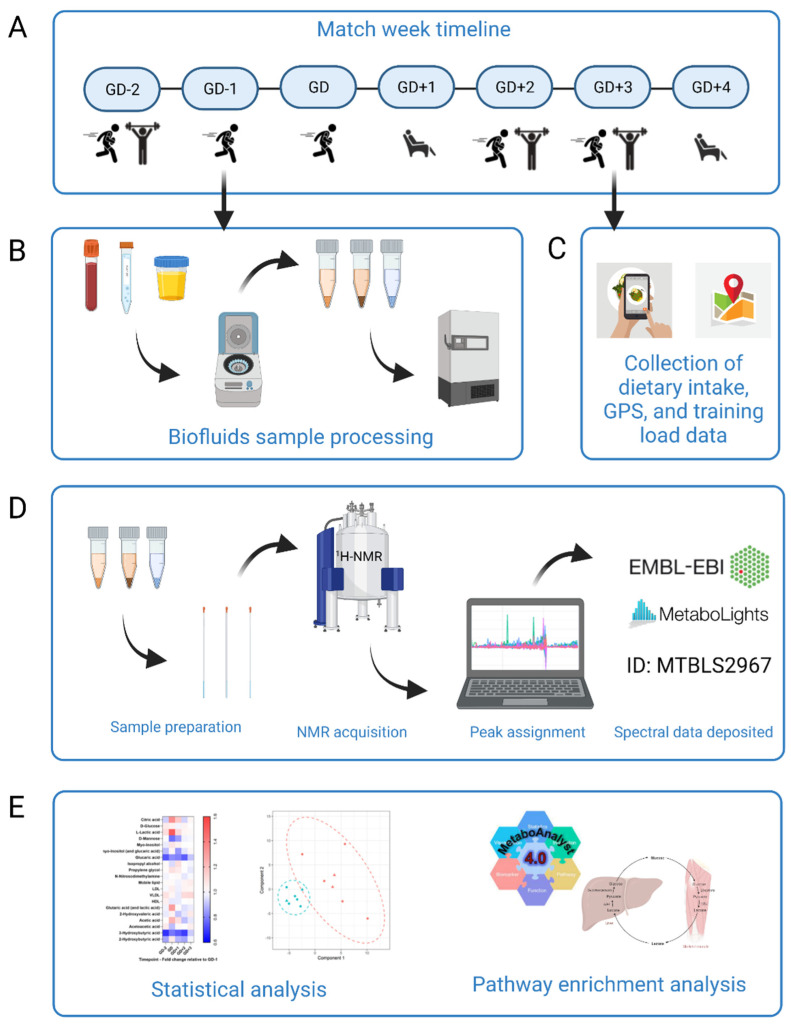
Schematic overview of the study design. (**A**) Participants (*n* = 7) began the study at GD-2 and completed a whole match week schedule of rugby specific sessions, resistance training and rest. (**B**) Biofluids sample processing. Participants provided samples of blood, urine, and saliva every morning apart from the GD sample which was taken immediately post-match play. Samples were processed immediately with timings rigorously repeated each day. Biofluids were then frozen for later analysis. (**C**) Dietary intake for all seven days using the Snap’n’Send method, with all GPS and load data were collated. This was all analyzed to further translate changes to the metabolome. (**D**) Sample preparation and analysis by ^1^H-NMR. Spectra were acquired and then peaks assigned using Chenomx. Full spectrum parameter sets are available with the data deposited at MetaboLights public repository (ID number MTBLS2967). (**E**) Statistical analysis. Univariate and multivariate data analysis was performed in R to elucidate key metabolites between all sample timepoints. MetaboAnalyst 4.0 pathway enrichment analysis was then carried out with those statistically significant and key discriminatory metabolites. Figure created using BioRender.

Taken together, our overarching objective is to capture the metabolic fluctuations that occur in response to elite rugby union match play prior to, and in the days after the cessation of the match, which will allow us to generate new hypotheses around athlete recovery after competitive match play (Figure 1). Moreover, this is the first research to investigate the blood, urine and saliva body fluids in an elite athletic population and will provide a novel view of collision-based team sport upon the metabolome. 

## 2. Results

This section will be divided in to the ‘acute’ changes to the metabolome analyzed utilizing the samples gathered immediately after match play, and the ‘recovery’ period which compares the fasted samples from the day preceding (GD−1) with the days after match play. Dietary intake was analyzed for every day to ensure specific metabolite appearance and pathways potentially related to macronutrient intake could be accounted for. 

### 2.1. Dietary Intake

Macronutrient and energy intakes are reported here as mean (±SD) for the week with a more detailed Table S1 od daily intakes available in the supplementary material. There was no difference in daily protein intake 2.39 ± 0.33 g/kg (*p* = 0.3743), and fat intake 1.16 ± 0.15 g/kg (*p* = 0.3666) across the match week. There were significant differences in carbohydrate intake across the days of the match week 3.17 ± 0.37 g/kg (*p* < 0.0001). Intake on GD−1 (4.32 ± 0.89 g/kg) and GD (5.62 ± 0.85 g/kg) were significantly higher than every other day and GD itself was higher than GD−1 (*p* = 0.0032). This pattern was mirrored in total energy with a mean intake of 3323 ± 630 kcal/day across the week.

### 2.2. Acute Changes to the Metabolome Post Match Play

Univariate analyses yielded four significant metabolites (alanine, citrate, and two unidentified saccharides) in the GD samples in serum. Multivariate analyses of all biofluids generated seven high quality PLS-DA models which identified the key discriminatory metabolites which went forward to pathway enrichment analyses and are displayed in Table 1. 

#### 2.2.1. Meeting the High Energy Demands of Elite Rugby Union

**Figure 2:**
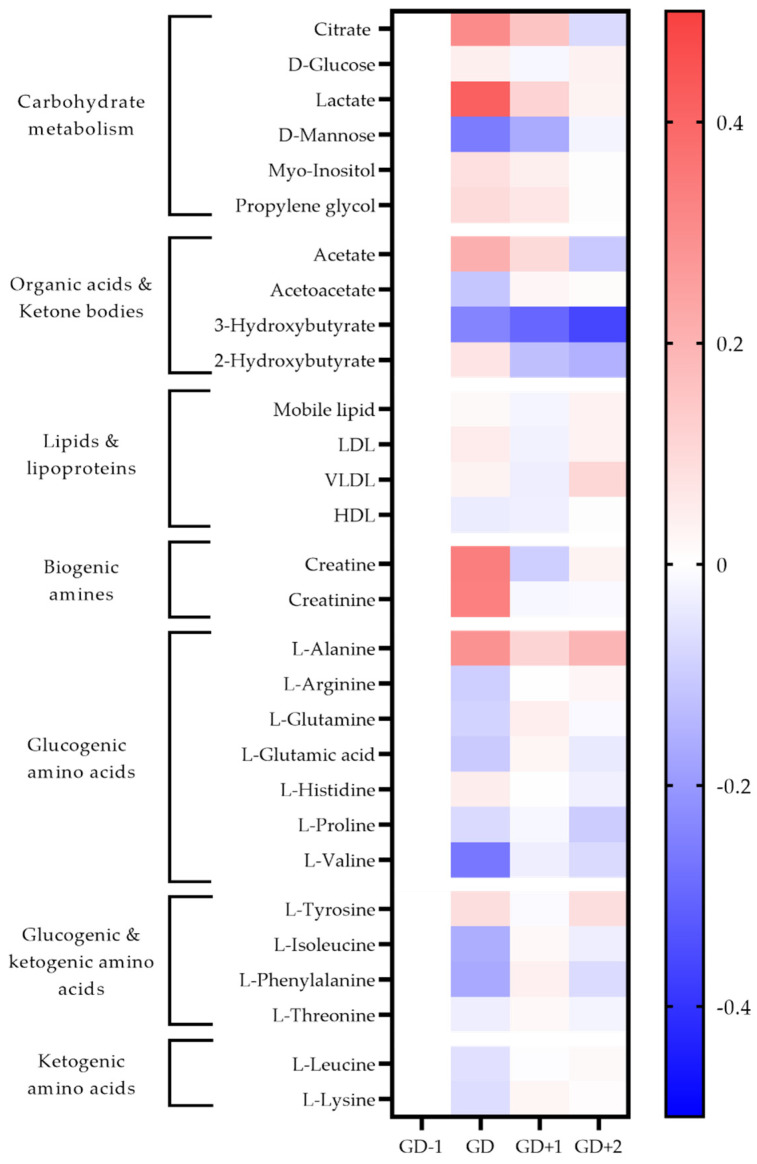
Heatmap of metabolites identified as significant via univariate analysis and as key discriminators between timepoints via PLSDA modelling in blood serum samples. The fold change calculated from the GD−1 sample is displayed as the natural logarithm to indicate an increase (greater than 0, red) or decreased (less than 0, blue).

**Figure 3:**
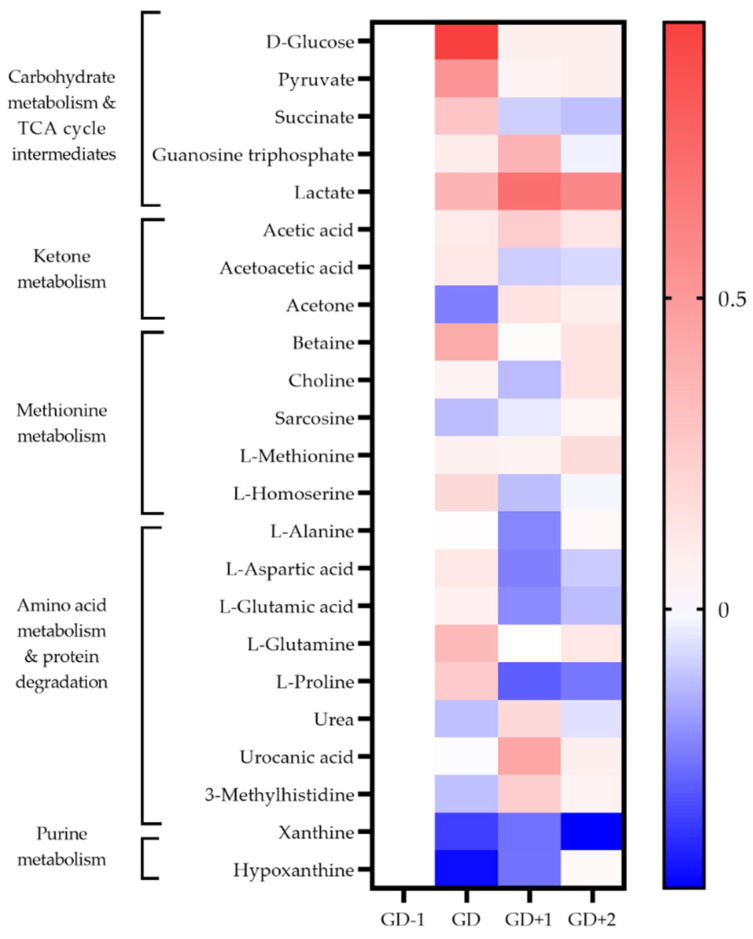
Heatmap of metabolites identified as key discriminators between timepoints via PLSDA modelling in saliva samples. The fold change calculated from the GD−1 sample is displayed as the natural logarithm to indicate an increase (greater than 0, red) or decreased (less than 0, blue).

The high energy demands of this sport are demonstrated by the inclusion of glycolysis, glucose-alanine cycle, and pathways associated with amino acid degradation being highly ranked in both serum and saliva samples immediately post-match (Table 1, Figure 2 and Figure 3). 

Two unidentified saccharides were significantly elevated in the GD serum samples (*p* = 0.030 and *p* < 0.0001) due to the ingestion of carbohydrate gels and drinks. Conversion of pyruvate into lactate ensures glycolysis can continue, explaining the serum lactate peak immediately post-match (Figure 2). Higher salivary pyruvate and lactate were also key discriminators in acute PLSDA models (Figure 3). Univariate analysis identified serum citrate (*p* = 0.032) as significantly increased at the GD sample, together with increases in serum acetate, and salivary TCA cycle intermediaries explaining the key pathways identified for energy provision during the match. 

Serum alanine, using univariate analysis was significantly increased at GD compared to GD−1 (*p* < 0.0001). The pathway enrichment for both serum and saliva had the glucose-alanine cycle ranked most highly, indicating gluconeogenesis as required to meet the total energy needs.

The key discriminatory metabolites associated with the ketone body metabolism pathway identified in saliva are succinic acid, acetoacetate, and acetone. Salivary acetone is much reduced compared to GD−1 levels, as are serum acetoacetic acid and 3-hydroxybutyrate. This indicates a reduced fatty acid oxidation during match play.

#### 2.2.2. Amino Acid Metabolism

**Figure 4:**
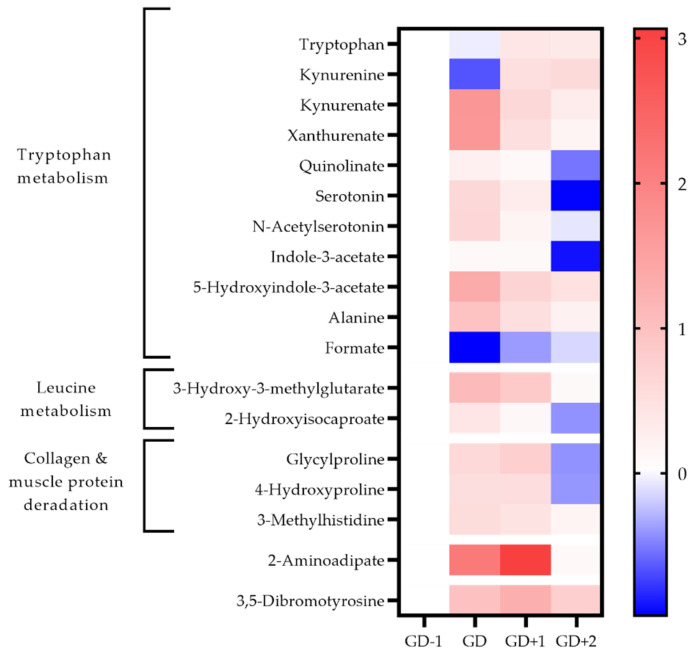
Heatmap of metabolites identified as key discriminators between timepoints via PLSDA modelling in urine samples. The fold change calculated from the GD−1 sample is displayed as the natural logarithm to indicate an increase (greater than 0, red) or decreased (less than 0, blue).

Both the urea cycle and ammonia recycling are highly ranked pathways in serum and saliva acutely post-match (Table 1). All serum amino acids apart from alanine, histidine and tyrosine are reduced in the GD samples (Figure 2). The metabolites of leucine; 2-hydroxyisocaproate, and 3-hydroxy-3-methylglutarate are also identified in urine to increase in the GD samples, further confirming the degradation of this branched chain amino acid (BCAA) (Figure 4). 

**Figure 5:**
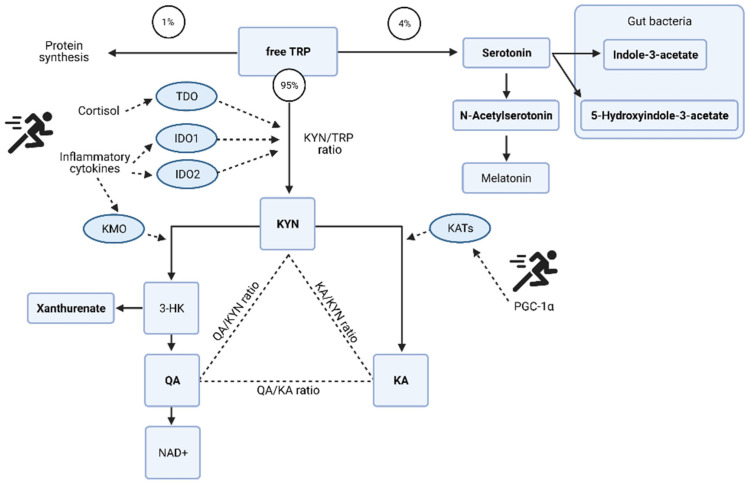
Potential mechanisms of tryptophan (TRP) metabolism and the kynurenine (KYN) pathway induced by exercise. Redrawn from [27] with additional information and the specific metabolites identified as key discriminators via PLSDA modelling in the acute post-match and recovery period. Kynurenate (KA); quinolinate (QA); oxidized form of nicotinamide adenine aminotransferase (NAD+); 3-hydroxykynurenine (3-HK); tryptophan 2,3-dioxygenase (TDO); indolamine 2,3-dioxygenase (IDO); kynurenine 3-monooxygenase (KMO); kynurenine aminotransferase (KATs); proliferator-activated receptor-gamma coactivator-1alpha (PGC-1α). Urinary metabolites identified via multivariate analysis are in bold. Figure created with BioRender.

The acute changes in the urinary metabolome identified tryptophan metabolism as the key pathway (Table 1). The changes in associated metabolites are visualized in Figure 4 and the potential mechanisms induced by exercise shown in Figure 5. There is an acute shift for downstream metabolites of the kynurenine pathway such as kynurenate (KA), quinolinate (QA), and xanthurenate to increase, with a marked reduction in kynurenine (KYN). 

#### 2.2.3. Acute Oxidative and Metabolic Stress

The organic acid 2-hydroxybutyrate peaks acutely post-match in serum. Whilst salivary purines, xanthine and hypoxanthine are at their lowest levels in the GD samples. 

### 2.3. Changes in the Metabolome in Recovery from Match Play

Univariate analysis identified one metabolite, alanine as significantly (*p* = 0.0019) increased at GD+2, in the recovery period. Multivariate analysis across all biofluids generated seven high quality models (ROC > 0.75) via PLSDA. The metabolites identified as key discriminators (VIP > 1) between samples were put forward for pathway enrichment analysis, the results of which are displayed in Table 2. 

#### 2.3.1. Amino Acid Metabolism

Levels of serum amino acids appear to normalize at GD+1 (Figure 2). Alanine remains above pre-match levels with citrate, lactate, and acetate. However, between the morning after the match at GD+1, and the GD+2 timepoint there is a shift denoted by pathways of glucose-alanine cycle, gluconeogenesis, aerobic glycolysis, BCAA degradation, and ammonia recycling being ranked highly in serum and saliva (Table 2, Figure 2 and Figure 3).

Serum levels of alanine are significantly higher again (*p* = 0.019) at GD+2 compared with GD−1, and the levels of all glucogenic amino acids are reduced. The ketogenic amino acids leucine and lysine remain at pre-match levels in blood serum. 

#### 2.3.2. Markers of Structural Protein Degradation

3-Methylhistidine (3-MH) is a key discriminatory metabolite in both urine and saliva (Figure 3 and Figure 4). Levels in both biofluids peak at GD+1 with elevated levels in urine at GD and in saliva at GD+2 also. Collagen metabolites, glycylproline and 4-hydroxyproline are both elevated in urine post-match and into the recovery period (Figure 4). 

#### 2.3.3. Metabolic and Oxidative Stress

Pathway enrichment of the key discriminatory metabolites between GD-1 and GD+2 also identified glutathione metabolism, and glycine & serine metabolism in serum, together with methionine metabolism in saliva (Table 2). Urinary 3,5-dibromotyrosine peaks in recovery at GD+1 and is above pre-match abundances in all comparison timepoints. 

#### 2.3.4. Fatty Acid Metabolism

Acetoacetate in serum normalizes in recovery with 3-hydroxybutyrate levels staying well below pre-match concentrations throughout. Serum lipoprotein fractions shift with reductions in HDL at GD and GD+1, whilst VLDL rises in recovery at GD+2. The urinary metabolome in recovery exhibits a 21-fold increase of 2-aminoadipic acid (2-AAA). 

### 2.4. Training and Match Demands

Table 3 and Table 4 present the training objectives for the match week together with physical metrics of load. Demonstrating that these athletes are exposed to a training load containing high-speed running (HSR), very high-speed running (VHSR), high velocity accelerations, and decelerations comparable to a match day, repeatedly in training. We can conclude they are accustomed to the volume and intensity of dynamic high-speed movements as per match play but not exposed to full collisions in training. 

## 3. Discussion 

This is the first research to investigate the metabolome across multiple biofluids in an elite athletic population and to provide characterization of the metabolic perturbations caused by competitive rugby union. The acute energy needs of this high intensity sport are met via glycolysis, the TCA cycle and gluconeogenesis. The recovery period after cessation of match play and prior to training recommencing sees a re-entry to gluconeogenesis, coupled with markers of oxidative stress, structural protein degradation, and altered fatty acid metabolism. This complex and integrated whole-body response allows us to discuss how best to fuel recovery after collision-based team sports for the first time.

A novel part of our exercise metabolomics research design was the simultaneous analysis of dietary intake to account for the influence of nutrition on the metabolomic responses to rugby match play. This was particularly important as we analyzed the metabolome beyond the previously reported 24 h time point post exercise [8]. Monitoring dietary intake is essential to ensure our data can be translated in to the world of applied sports nutrition [28]. Carbohydrate intake of the players here was periodized with training load influenced by the ‘Fuel for the work required’ paradigm framework [29], and prioritising a high intake at GD−1 to ensure high glycogen levels for performance [30]. A target of 6 g/kg carbohydrate may be appropriate on GD−1 to ensure sufficient glycogen and may be more appropriate than 3 g/kg [30]. Carbohydrates were also consumed during the match as per ingestion guidelines of 60 g/h [31] in the form of drinks and gels, which yielded the two significant unidentified saccharides in serum. These athletes did not meet the upper target of carbohydrate on GD−1 but at 4.32 ± 0.89 g/kg we would not expect this combined with the high intake on GD pre-, and peri-match to have limited glucose availability. Nevertheless, this novel insight provides further evidence as to the importance of carbohydrates for performance. Glycolysis, together with the TCA cycle and glucose-alanine cycle are the predominant pathways accounting for energy production during match play. Serum acetate levels do peak at GD and the beta-oxidation of fatty acids could contribute to this. However, there is no accumulation of acetoacetate or 3-hydroxybutyrate in serum which would indicate the saturation of β-oxidation as seen previously in prolonged endurance exercise [32]. This combined with reduced saliva acetone leads us to conclude the high intensity nature of elite rugby union reduces the utilization of fatty acids for energy provision. 

Rather, pyruvate conversion to lactate ensures glycolysis can continue, pyruvate can also enter the TCA cycle to further generate ATP [33]. Lactate is transported to the liver and converted to glucose to be transported back to the muscle or peripheral tissues [34]. Pyruvate can also be converted to alanine in the muscle via the aminotransferase enzyme, in turn also converting glutamate to alpha-ketoglutarate [33]. The significant increase in serum alanine as reported here, has previously been observed within 30 min of exercise [8], specifically after high intensity training (HIT) rather than isoenergetic, moderate intensity exercise [9]. This gluconeogenesis is accompanied by amino acid degradation and the upregulation of the urea cycle and ammonia recycling, evidenced in serum and saliva. It is important to note that the origin of these amino acids is unclear, as they may be entering the bloodstream via the gut as the GD samples were not fasted and protein sources were consumed at breakfast and pre-match meal. Glutamate in the muscle can be converted to glutamine, transported in the blood to the liver to be converted back into glutamate to aid in the ammonia recycling to supply the alanine aminotransferase reaction for alanine to pyruvate conversion, and then the ammonia feeds into the urea cycle for detoxification [34]. The rise in serum tyrosine levels can be explained by the conversion of phenylalanine into tyrosine during exercise [35]. Amino acid metabolism during prolonged exercise appears in the early phases to create a net consumption of glutamate from the muscle to replenish TCA cycle intermediates [36]. The formation of α-oxoglutarate, succinyl-CoA, and oxaloacetate from glutamate, valine, and isoleucine are mechanisms for anaplerosis in exercise which may explain a number of the reductions in serum amino acids witnessed here [35]. 

The urinary metabolome further supports how the system copes with the high energy demands. Post-match there was a drop in tryptophan and an increase in serotonin together with the downstream products of the kynurenine (KYN) pathway, the neuroprotective kynurenate (KA), and quinolinate (QA) which is associated with excitotoxicity [37]. The de novo synthesis of NAD+ can occur from QA, and may be a crucial step in the high energy demands of exercise to improve energy homeostasis [38] explaining the marked shift in the QA:KYN ratio post-match. 

Overall, the intensity and duration of elite rugby union match play cause acute metabolite perturbations indicative of both oxidative and metabolic stress whilst fulfilling the high energy demands. The reduced salivary purine profiles due to oxidative stress have been observed post exhaustive exercise in male athletes [39]. The increase in serum 2-hydroxybutyrate post-match is indicative of the cumulative stress upon energy systems [40] and may be due to increased catabolism of l-threonine [41] and glutathione synthesis [42] in response to oxidative stress. Transient increases have also been witnessed after HIT exercise [9] and proposed as a marker of dysglycemia [43]. In non-diabetic populations it is an early biomarker for both insulin resistance and impaired glucose regulation [44]. This is particularly interesting as the changes to the metabolome in recovery that follow, implicate changes in glucose regulation and gluconeogenesis. The pathways of glutathione metabolism, and glycine and serine metabolism are ranked highly in recovery, with reductions in serum l-threonine indicative of increased demands for hepatic glutathione production [42] as this oxidative stress continues in the days after match play.

As we have shown previously [6], in training, these athletes are exposed regularly to the high intensity activities of the sport, but without full collisions. In the days following match play they are therefore recovering from muscle damage due to unaccustomed activities and the collisions inherent with tackling, carrying, and contesting the ball which result in EIMD and IIMD [4], and it is the resulting metabolic perturbations we are examining herein. Whatever the primary mechanism of ultrastructural damage, the cascade of events comprising the secondary mechanism is triggered by an acute inflammatory response due to the action of immune cells such as neutrophils and macrophages [45]. 

The increased cytokine concentrations of IL-6 [24,25], IL-8 and IL-10 [26] have been profiled in this population post-match play. The realization of this potential inflammatory response on the urinary metabolome is in the tryptophan metabolism pathway. Pro-inflammatory cytokines interleukin (IL)-6 and tumor necrosis factor (TNF)-α [46,47] cause an initial shift in the KYN pathway, but then in recovery as the need for de novo synthesis of NAD+ abates, QA levels reduce and TRP, KYN, and KA remain elevated above pre match levels. The continued conversion of KYN to KA can be due to peroxisome proliferator-activated receptor-γ family of transcriptional coactivators, specifically PGC-1α, whose expression is induced by exercise and plays a crucial role in skeletal muscle adaptation [48]. 

The inflammatory activity may also be responsible for the reductions in serum HDL levels at GD and GD+1, whilst VLDL rises in the GD+2 and GD+3 samples are also associated with cytokine activity [49]. The increase in VLDL production and secretion is a result of: increased hepatic fatty acid synthesis, increase in transport of fatty acids to the liver and a decrease in fatty acid oxidation in the liver [49]. This potential change in fatty acid oxidation is supported by the large increase in 2-AAA in the urinary metabolome peaking at GD+1. This product of lysine degradation and predictive marker of type-2 diabetes in normoglycemic individuals [50], is associated with adipogenesis, and it is proposed that in early insulin resistance it upregulates insulin secretion to maintain normal glucose homeostasis which can induce abnormal gluconeogenesis [51]. We therefore propose that fatty acid oxidation is impaired in the recovery period from rugby union match play due to the inflammatory response to muscle damage.

Examination of the immune response post elite rugby union has revealed significant increases in total leukocytes, specifically neutrophils and monocytes peaking acutely post-match play and remaining significantly elevated compared to baseline measures [24,25]. Increases in lymphocytes, specifically significant increases in CD4^+^ have also been witnessed at the GD+1 timepoint [24]. The increased urinary 3,5-dibromotyrosine observed herein, peaking at GD+1 is indicative of eosinophil activity and may indicate protein oxidation of injured tissue [52]. Any reducing or blocking of this initial immune cell response can interfere in regeneration and subsequent adaptive remodeling of muscle tissue [45]. Neutrophils respond to stimuli by enhancing their glucose uptake and increasing expression of glucose transporters suggesting a functional dependence of glucose in modulating their function, especially phagocytic events [53]. Activated CD4^+^ and CD8^+^ T cells both display elevated glycolysis in vivo, critical for rapid growth and proliferation [54]. 

The reduced serum levels of glucogenic amino acids in recovery, coupled with the pathways identified as highly ranked in saliva and serum lead us to propose that this requirement for glucose due to the secondary response to muscle damage has not been met via dietary intake. Similar dietary carbohydrate periodisation has been reported previously [55] with a mean weekly intake of 3.4 g/kg compared to our 3.2 g/kg here, within the intakes across the literature for this population [56]. The intake of carbohydrate on GD+1 was below the mean for the week at 2.9 g/kg, again like the earlier work in an elite rugby union population in-season reporting 3.1 g/kg [55]. This earlier work recorded similar energy intakes to those observed here and reported a predicted balance of energy utilizing wearable devices to measure TEE [55]. There are limitations to these measures of TEE, but our findings here suggest that energy availability generally may not be as crucial for recovery as carbohydrate availability specifically. Revisiting our previous work we can calculate the significant increase in fasted carbohydrate oxidation at rest to be from 228 g/day the morning prior to match play, up to 319 g/day at GD+2 [6]. We propose increasing dietary carbohydrate intake at GD+1 to account for this increased resting requirement, normal daily activity and any light recovery modalities would result in a significant increase on the intake observed here and in previous observations of this population. The hypothesis if this carbohydrate requirement is met, being a reduction in amino acid degradation and gluconeogenesis activity. 

The insight provided here in the recovery period, utilizing fasted samples demonstrates the potential ramifications of amino acid degradation to provide fuel for this immune response. 3-Methylhistidine (3-MH) has been purported as a marker of myofibrillar protein degradation [57] leading to its release, and excretion in urine as it cannot be re-utilized [58]. Salivary 3-MH has been observed after basketball match play [19] and in male soccer players identified as fatigued after three consecutive days of match exposure [17]. Here urinary and salivary 3-MH are key discriminatory metabolites with increases at GD+1, and in saliva at GD+2 also, compared with pre-match values. Elevated urinary levels of the collagen metabolite hydroxyproline have been recorded after eccentric activities [59] with the question of whether this is due directly to the unaccustomed exercise or the ensuing inflammatory response still to be answered. This evidences the potential that the degradation of amino acids to meet glucose requirements causes a loss to the structural integrity of connective tissue and muscle protein in the recovery period. 

Whilst ergogenic aids and functional foods may provide acute nutritional strategy for muscle damage [60], recent work from our group demonstrated concentrated polyphenol rich supplementation provided no enhanced benefit compared to dietary intake of whole food sources [26]. It would seem most pertinent therefore that to maximize the immune cell response and allay the inflammation, allowing efficient regeneration and remodeling of damaged tissue, glucose provision should be the priority. Future work should investigate interventions prescribing higher carbohydrate intakes at GD+1. Providing athletes with meals to consume 5–6 g/kg body mass for GD+1. Examination of the gluconeogenesis metabolic pathways, together with markers of protein degradation and adipogenesis would provide insight as to whether these intakes higher than previously recorded in this population, allay potential negative effects upon recovery, reducing muscle protein and connective tissue degradation. Stratification of player position was also not possible in this sample size and future targeted metabolite studies that consider positional collision activities will test the hypotheses presented here further.

In conclusion, novel insight is provided into how energy systems cope with demand acutely around rugby match play, but also in the recovery days prior to training recommencing. Rather than the availability of energy being the priority, a potential reduction in the ability to oxidize fatty acids, coupled with glucogenic amino acid degradation with upregulated gluconeogenesis leads us to propose that the effective recovery from muscle damaging collisions during elite rugby union match play, is dependent upon the availability of glucose. If sufficient glucose from dietary carbohydrate can be provided in the day after match play this may facilitate the regeneration and remodeling of damaged muscle tissue optimally and prevent the oxidation of amino acids which may also be crucial for the retention of muscle mass and connective tissue integrity. This would translate into the periodisation of carbohydrate throughout the competitive microcycle in contact sports to be re-evaluated to prioritize carbohydrate in recovery whilst balancing energy intake to maintain optimal body composition and performance throughout a season. Should we expand the ‘Fuel for the work required’ paradigm in collision-based team sports to include ‘Fuel for the damage induced’? 

## 4. Materials and Methods

### 4.1. Participants and Research Design

Following ethical approval and informed consent, seven healthy elite rugby union players were recruited for this study, all members of an English Premiership squad (mean ± SD, age; 22.0 ± 2.7 years, body mass; 102.5 ± 13.7 kg). All participants gave full written consent prior to commencing the study. Ethical approval (19/SPS/039) was granted by the university research ethics committee at Liverpool John Moore’s University (Liverpool, UK). 

Venous blood, urine, and saliva samples were collected throughout a competitive match week during the early part of the competitive season. Time points throughout the study are described relative to game day (GD) using +/−symbols for the days preceding (−) and days after (+) GD. Due to the timing of selection defining when recruitment could occur, the first measurement was taken at GD−2. Figure 1 shows the study design and workflow.

### 4.2. Training and Match Demands

Internal loads for each training day and the game day were assessed by the session rating of perceived exertion (sRPE) using a modified Borg scale [61]. This RPE of the training session was multiplied by the training duration to calculate a player load in arbitrary units (sRPE [61]. External demands of all rugby training sessions and match play were recorded using micro-technological units worn by players containing GPS (10 Hz) and accelerometer (100 Hz) (Catapult Innovations, Melbourne, Australia). Data were downloaded and analyzed using Catapult Sprint software (Catapult Innovations,). The total distance covered, number of high-speed efforts (>60% positional average) and the number of very high-speed efforts (>80% individual average) were recorded [62,63]. The GPS sampling frequency of 10 Hz is the most reliable in team sports measuring high-speed running activities [64]. Internal and external loads were recorded for both training and match play throughout the week. Table 3 and Table 4 detail the physical content of the match week. 

### 4.3. Dietary Intake

Dietary intake was recorded using the participants mobile phone device incorporating the ‘Snap’n’Send’ method [65]. The athletes were educated in their nutrition requirements. A wide range of meals and snacks designed by the team nutritionist were provided at the training facility. Their choices and portions in the club dining facility and whilst away from there were self-selected. The dietary analysis software Nutritics (Nutritics Ltd., Dublin, Ireland) was used by a registered sports and exercise nutritionist (SENr) to analyze food intake over the match week. Analyzing dietary intake has also allowed us to account for metabolites associated with the ingestion of foods and any dietary supplements in our conclusions [66].

### 4.4. Biofluid Sample Collection

Biofluid samples were collected every morning apart from the GD time point when the samples were collected within 30 min of the final whistle post-match play. Post-waking, participants reported to the training ground in a fasted state and provided a venous blood, urine, and saliva sample. A fasted sample was not taken on the morning of the match as this would likely reduce the number of players willing to participate and be perceived as too much disturbance to their routine. It should be noted authors have also refrained from this due to concerns around metabolite changes caused by the stress of venipuncture and have used the day prior as the baseline measure [32]. 

Whole blood samples (10 mL) were drawn from a superficial vein located in the antecubital fossa of the forearm using standard venipuncture techniques. Samples were collected using serum tubes (Vacutainer Systems, Becton Dickinson, Franklin Lakes, NJ, USA) which did not contain clotting gels or additives as these may interfere with metabolomics analysis [67]. Samples clotted at room temperature (18–22 °C) for 40 min prior to centrifugation at 1600× *g* for 15 min. Urine was collected and centrifuged at 1600× *g* for 15 min in 15 mL urine centrifuge tubes (Sarstedt, Leicester, UK) which contained no citrate or other stabilizers. Saliva samples were collected using the previously validated Salivette swabs (Salivette Sarstedt, Nubrecht, Germany) without additives, centrifuged at 1500× *g* for 15 min [15]. 

All samples were aliquoted into 2 mL cryovials (Fisherbrand, Loughborough, UK) and immediately frozen at −24 °C. Upon completion of the research study all samples were transferred to −80 °C for longer term storage, prior to metabolomics processing and spectral analysis. The samples were used within the nine month guide for storage best practice [68]. The time taken to process the samples each day was recorded and rigorously replicated to ensure reduced between-sample variability as a result of sampling and the highest possible sample quality [69].

### 4.5. NMR Spectroscopy

NMR spectroscopy was preferred here due to its ability to attenuate the signals of higher molecular weight metabolites [70], confidently identify and examine the more abundant compounds of all three biological fluids [71] whilst being non-destructive and highly reproducible [72], despite being less sensitive than mass spectrometry techniques [70]. 

### 4.6. NMR Sample Preparation

Aliquots were thawed and 500 μL of serum was diluted to a final volume containing 50% (*v/v*) serum, 40% (*v/v*) dd ^1^H_2_O (18.2 MΩ), 10% (*v/v*) 1 M PO_4_^3−^ pH 7.4 buffer (Na_2_HPO_4_, VWR International Ltd., Radnor, PA, USA and NaH_2_PO_4_, Sigma-Aldrich, Gillingham, UK) in deuterium oxide (^2^H_2_O, Sigma-Aldrich) and 1.2 mM sodium azide (NaN_3_, Sigma-Aldrich). Samples were vortexed for 1 min, centrifuged at 13,000× *g* at 4 °C for 2 min and 600 μL transferred into 5 mm outer diameter NMR tubes (Bruker, Coventry, UK).

Urine & saliva samples were thawed at room temperature before addition of 500 μL to 500 μL of 1 M phosphate buffer (Na_2_HPO_4_ and NaH_2_PO_4_) at pH7.4 with 20% ^2^H_2_O, 200 μM TSP and 2.4 mM sodium azide. The samples were vortexed for 30 s prior to 5 min centrifugation at 21,500× *g* and 4 °C before transferring 600 μL of sample to Bruker SampleJet 5 mm (outer diameter) NMR tubes. The final concentration in the NMR tube was 50% urine or saliva, 10% ^2^H_2_O, 1.2 mM sodium azide and 100 μM TSP.

### 4.7. NMR Acquisition

All spectra were acquired using a 700 MHz Bruker Advance IIIHD spectrometer equipped with a TCI cryoprobe and chilled Sample-Jet autosampler. Blood serum and saliva samples were analyzed via 1D ^1^H NMR standard experiment for selective observation of low molecular weight components with optimal water suppression was acquired, pulse sequence is vendor supplied Carr-Purcell-Meiboom-Gill (CPMG) sequence (cpmgpr1d, Bruker). CPMG spectra were acquired with 32 transients a 30 ppm spectral width, 64 k points, 9.6 ms echo time and a 3.1 s acquisition time and a 4 s interscan delay. Blood serum spectra were acquired at 37 °C in accordance with best practice [69].

Urine and saliva 1D ^1^H-NMR spectra were acquired at 25 °C to facilitate analysis via Chenomx Standard library. Urine spectra were analyzed via 1D ^1^H-NMR standard pre-saturation experiment for optimal water suppression (vendor supplied noesypr1d). NOE spectra were acquired with 32 transients a 25 ppm spectral width, 96 k points, 2.7 s acquisition time and a 4 s interscan delay. 

Full spectrum parameter sets are available with the data deposited at MetaboLights public repository ID number MTBLS2967 [73].

### 4.8. Spectral Processing and Annotation

All spectra were analyzed to ensure conformity with the recommended minimum reporting standards set out by the Metabolites Standard Initiative (MSI) [74,75]. Serum spectra were aligned to glucose anomeric peak at 5.24 ppm whereas urine and saliva spectra were aligned to the TSP peak at 0 ppm. spectra underwent automated data processing, Fourier transformation and phasing carried out in Topspin v3.6 software using standard Bruker routines (apk0.noe).

Serum and saliva spectral peaks were annotated using a combination of Chenomx standard spectra and in house metabolite libraries with pattern files produced for both biofluids to enable spectral binning. Serum spectra were integrated into 160 bins with 104 (65%) annotated corresponding to 38 metabolites and 56 unknown metabolite bins. Saliva spectra were integrated into 251 bins with 134 (53%) annotated corresponding to 82 metabolites and 117 unknown metabolite bins. Spectra were binned or bucketed per peak into a matrix of metabolite peak intensities using tameNMR. Bin annotation and binned data is available with the dataset in Metabolights (MTBLS2967).

Chenomx v8.2 software was used to perform metabolite annotation on the individual urine spectra using automated fit all metabolites routine. Of the 251 metabolites annotated in Chenomx for the 117-sample set 46% (13,696 values) were missing. To provide a missing value estimation for this dataset metabolites with too many missing values <50% were removed—this reduced the number of annotated metabolites to 131. The remaining missing values were replaced by an estimate of the limit of detection corresponding to 0.2 of the minimum positive value of each variable. Manual confirmation of identities where possible to in-house standards for metabolite peaks found to be significantly variable. 

### 4.9. Data Analysis

Univariate and multivariate analyses was performed using R (Version 3.6.1, The R foundation for statistical computing). The scripts used were provided by the Computational Biology facility at the University of Liverpool (UK). Prior to univariate analysis, PQN normalization was performed as this is reported to be the most robust method in the analysis of complex biofluids [76,77]. Further to this, data were scaled and mean-centered prior to multivariate analysis. Pareto scaling with mean centering was performed as the preferred method to ensure the ability to identify small biologically significant variations in metabolites [78]. Univariate analysis was performed on the PQN normalized spectra across the week using a one-way ANOVA using a Benjamini-Hochberg (FDR) method of multiple correction at a significance level of *p* < 0.05. A post-hoc Tukey analysis provided pairwise comparisons of specific time points. Partial least squares discriminant analysis (PLS-DA) was used for multivariate analysis, specifically identifying differences in metabolites between time points. Models generated via PLS-DA were evaluated using a random 30% of the data held back to test the model and produce receiver operator characteristic (ROC) scores. Specific metabolites within each model were only used for further analysis if the Variable Importance in Projection (VIP) scores were above 1.00 and ROC scores ≥0.75. 

Pathway analysis was performed using MetaboAnalyst (Enrichment analysis, version 4.0, metaboanalyst.ca) [79]. Ranked *p* values are reported here where *p* < 0.05 without adjustment. Only specific metabolites identified using PLS-DA between time-points were entered into the enrichment analysis. Heatmaps were generated incorporating metabolites identified from univariate and multivariate analysis. The fold change is reported relative to GD-1 and then natural log is displayed.

Statistical analysis of the physical loads and dietary intake were performed using SPSS (Version 26 for Windows, SPSS Inc., Chicago, IL, USA) and GraphPad Prism (Version 8.4.3 for Windows, GraphPad Software, San Diego, CA, USA). All data are presented as mean (±SD), and a one-way repeated measures ANOVA was used to compare all measures across the week. The test of within subjects’ effects provided values for Mauchly’s test for sphericity. If this was violated, then a Greenhouse-Geisser correction was used. The difference between means was tested at a significance level of *p* < 0.05. A Tukey correction post hoc was used to compare specific time points when the ANOVA revealed a significant difference between measures over the week. 

## Figures and Tables

**Table 1:** Metabolites identified as key discriminators between samples collected immediately post-match play (GD) and the GD−1, GD+1, and GD+2 timepoints via PLSDA modelling were then put forward for pathway enrichment analysis using MetaboAnalyst 4.0. The unadjusted ranked *p*-values are displayed here.

Biofluid	Acute Pathways	Timepoint Comparison (Unadjusted *p*-Value)			Metabolites Included
		GD−1 vs. GD	GD vs. GD+1	GD vs. GD+2	
Blood Serum	Glucose-Alanine Cycle	0.0019	0.0019	0.0025	d-Glucose, l-Glutamic acid, l-Alanine
	Urea Cycle	0.0022	0.0193	0.0032	l-Glutamic acid, l-Alanine, l-Arginine, l-Glutamine
	Warburg Effect (aerobic glycolysis)	0.0050	0.0050	0.0075	Citrate, d-Glucose, l-Glutamic acid, Lactate, l-Glutamine
	Valine, Leucine, and Isoleucine Degradation	0.0301	0.0301	0.0087	l-Glutamic acid, l-Isoleucine, l-Leucine, l-Valine, Acetoacetate
	Phenylalanine and Tyrosine Metabolism		0.0175	0.0225	Acetoacetate, l-Glutamic acid, l-Tyrosine
	Arginine and Proline Metabolism			0.0272	Creatine, l-Glutamic acid, l-Proline, l-Arginine
	Ammonia Recycling	0.0252	0.0252	0.0321	l-Glutamic acid, l-Histidine, l-Glutamine
Urine	Tryptophan Metabolism	0.0051		0.0132	Formic acid, l-Alanine, Serotonin, l-Kynurenine, Kynurenic acid, 5-Hydroxyindoleacetic acid, Xanthurenic acid, l-Tryptophan, N-Acetylserotonin
Saliva	Urea Cycle		0.0254		l-Glutamic acid, l-Alanine, l-Aspartic acid, l-Glutamine
	Glucose-Alanine Cycle		0.0129		d-Glucose, l-Alanine, l-Glutamic acid
	Ketone Body Metabolism		0.0129	0.012	Acetoacetic acid, Succinic acid, Acetone
	Ammonia Recycling		0.0353		l-Glutamic acid, l-Aspartic acid, Urocanic acid, l-Glutamine

**Table 2:** Results of pathway enrichment analysis using the key discriminatory metabolites generated via PLSDA modelling. The unadjusted ranked *p*-values are displayed here.

Biofluid	Recovery Pathways	Timepoint Comparison (Unadjusted *p*-Value)			Metabolites Included
		GD−1 vs. GD+1	GD−1 vs. GD+2	GD+1 vs. GD+2	
Blood Serum	Glucose-Alanine Cycle	0.0182	0.0005	0.0182	d-Glucose, l-Alanine
	Galactose Metabolism	0.0224			d-Glucose, d-Mannose, Myo-inositol
	Glutathione Metabolism		0.0314		l-Glutamic acid, l-Alanine
	Glycine and Serine Metabolism		0.0420		l-Glutamic acid, l-Alanine, l-Threonine
	Valine, Leucine, and Isoleucine Degradation		0.0438		l-Glutamic acid, l-Isoleucine, l-Valine.
	Pyruvate Metabolism			0.0414	Acetic acid, Lactate, Propylene glycol
Urine	Tryptophan Metabolism			0.0349	Indoleacetic acid, Quinolinic acid, Serotonin, l-Kynurenine, Kynurenic acid, l-Tryptophan.
Saliva	Urea Cycle	0.00333			l-Glutamic acid, l-Alanine, l-Aspartic acid, Urea, Glutamine
	Aspartate Metabolism	0.00772	0.0309		Acetic acid, l-Glutamic acid, l-Aspartic acid, l-Glutamine, Guanosine triphosphate
	Glucose-Alanine Cycle	0.0104		0.00828	d-Glucose, l-Glutamic acid, l-Alanine, Pyruvic acid
	Ammonia Recycling	0.0275	0.0229		l-Glutamic acid, l-Aspartic acid, Urocanic acid, l-Glutamine
	Methionine Metabolism		0.0147		Betaine, Choline, Sarcosine, l-Methionine, l-Homoserine
	Warburg Effect		0.0477		d-Glucose, l-Glutamic acid, Succinic acid, l-Glutamine
	Gluconeogenesis			0.0282	d-Glucose, l-Lactic acid, Pyruvic acid, Guanosine triphosphate

**Table 3:** The in-season training schedule including session content and physical objectives.

Time Point	GD−3	GD−2	GD−1	GD	GD+1	GD+2	GD+3	GD+4
Purpose	Rest & Recovery	Intensity Execute tactical specifics at high intensity	Team Run Low intensity rehearsal of game plan	Match Play Maximal physical performance	Rest & Recovery	Installation Tactical learning	Overload run volume	Rest & Recovery
Resistance Training Content	None	Upper Limb Strength (45 min)	None	None	None	Lower Limb Strength (45 min)	Upper Limb Strength (45 min)	None
Physical Rugby Content	None	Specific Game Prep (45 min) Unit Split (20 min)	Execution of specific game plan at a low intensity (35 min)	Individual & Team prep. Rugby Match Play (80 min).	None	Low intensity attack shapes and defensive system installation. (50 min)	High Intensity throughout rugby specific drills. (75 min)	None

**Table 4:** In-Season physical metrics from training sessions and game day throughout the match week. * Significant difference in pairwise comparison with Gameday (GD) metrics after one-way repeated measures ANOVA and Tukey post-hoc correction.

Time Point	GD−2	GD−1	GD	GD+2	GD+3	ANOVA (*p*-Value)
Player Load (sRPE x Time)	600.71 ± 69.72	78.00 ± 13.90 *	533.14 ± 120.32	253.57 ± 173.00 *	512.14 ± 211.79	*p* < 0.0001
HSR Distance (m)	164.00 ± 71.65	72.57 ± 25.13 *	198.43 ± 80.05	100.43 ± 86.42	150.29 ± 97.21	*p* = 0.0327
HSR Efforts (*n*)	13.57 ± 5.07	7.00 ± 2.27	12.57 ± 3.42	7.57 ± 5.80	8.57 ± 5.04	*p* = 0.0513
VHSR Distance (m)	17.00 ± 13.28	0.57 ± 1.40	16.29 ± 21.62	4.86 ± 7.85	17.86 ± 13.14	*p* = 0.0733
VHSR (*n*)	1.71 ± 1.28	0.14 ± 0.35 *	1.29 ± 0.88	0.43 ± 0.73	1.29 ± 1.03	*p* = 0.0007
Accelerations > 3 ms (*n*)	9.00 ± 2.45	2.00 ± 1.41 *	6.43 ± 0.90	2.71 ± 3.15	3.14 ± 3.31	*p* < 0.0001
Decelerations > 3 ms (*n*)	10.29 ± 6.94	3.29 ± 1.67	10.71 ± 5.75	4.29 ± 2.96	5.14 ± 4.52	*p* = 0.0289

## Data Availability

Full spectrum parameter sets are available with the data deposited at MetaboLights public repository ID number MTBLS2967.

## Supplementary Materials

The following are available online at https://www.mdpi.com/article/10.3390/metabo11080544/s1, Table S1: Macronutrient intake for each day of the match week displayed relative to body mass (g/kg) and overall energy intake as total kilocalories (kcal).
