# Supplementary material for: “Fuel for the Damage Induced”: Untargeted Metabolomics in Elite Rugby Union Match Play
Source: Metabolites. 2021 Aug 17;11(8):544. doi: 10.3390/metabo11080544 (PMC8400368; doi:10.3390/metabo11080544)
Supplement: Supplementary file 1 [file metabolites-11-00544-s001.zip › metabolites-1339569-supplementary.pdf]

Supplementary Information

The information supplied below is supplementary to the section 2.1 in the main manuscript relative to dietary intake of the participants during the study period.

Table S1. Macronutrient intake for each day of the match week displayed relative to body mass (g/kg) and overall energy intake as total kilocalories (kcal). *Denotes significantly lower than GD, † denotes significantly lower than GD-1 after one-way repeated measures ANOVA and Tukey post-hoc correction.

Time Point	GD-2	GD-1	GD	GD+1	GD+2	GD+3	GD+4	ANOVA (p-value)
**Carbohydrate (g/kg)**	2.52 ±0.30*†	4.32 ±0.89*	5.62 ±0.85	2.93 ±0.64*†	2.11 ±0.42*†	2.42 ±0.51*†	2.25 ±0.68*†	p<0.0001
**Protein (g/kg)**	2.55 ±0.39	2.37 ±0.48	2.20 ±0.24	2.15 ±0.62	2.62 ±0.30	2.45 ±0.30	2.37 ±0.85	p=0.3743
**Fat (g/kg)**	1.09 ±0.33	1.17 ±0.18	1.05 ±0.35	1.34 ±0.28	1.04 ±0.24	1.19 ±0.11	1.25 ±0.40	p=0.3666
**Energy (kcal)**	3042 ±326*†	3770 ±235	4288 ±624	3272 ±379*	2856 ±151*†	3060 ±216*†	2971 ±625	p=0.0021
